# Impact of measurable residual disease in combination with CD19 on postremission therapy choices for adult t(8;21) acute myeloid leukemia in first complete remission

**DOI:** 10.1002/cam4.7074

**Published:** 2024-03-08

**Authors:** Xi Jia, Naying Liao, Sijian Yu, Huan Li, Hui Liu, Haiyan Zhang, Jun Xu, Yunqian Yao, Han He, Guopan Yu, Qifa Liu, Yu Zhang, Pengcheng Shi

**Affiliations:** ^1^ Department of Hematology, Nanfang Hospital Southern Medical University Guangzhou China; ^2^ Clinical Medical Research Center of Hematological Diseases of Guangdong Province Guangzhou China

**Keywords:** allogeneic stem cell transplantation, CD19, chemotherapy, MRD, t(8;21) AML

## Abstract

**Background:**

The post‐remission therapy (PRT) choices for adult t(8;21) acute myeloid leukemia (AML) in first complete remission (CR1) need to be further explored.

**Aims:**

We aimed to investigate the impact of measurable residual disease (MRD) combined with CD19 on PRT choices for adult t(8;21) AML in CR1.

**Methods:**

A total of 150 t(8;21) AML patients were enrolled, including 67 underwent chemotherapy (CMT) and 83 allogeneic hematopoietic stem cell transplantation (allo‐SCT) as PRT in CR1. Subgroup analyses were performed according to MRD level after three cycles of chemotherapy combined with CD19 expression.

**Results:**

Multivariate analysis indicated MRD^high^ after three courses of treatment (HR, 0.14 [95% CI, 0.03–0.66]; *p* = 0.013) and CD19 negativity (HR, 0.14 [95% CI, 0.02–0.96]; *p* = 0.045) were risk factors for relapse, while allo‐SCT was protective factor for relapse (HR, 0.34 [95% CI, 0.15–0.75]; *p* = 0.008). Grouped by MRD after three courses of chemotherapy, allo‐SCT had lower CIR (*p* < 0.001) and better OS (*p* = 0.003) than CMT for MRD^high^ patients, CMT showed a higher CIR (35.99% vs. 15.34%, *p* = 0.100) but comparable OS (*p* = 0.588) than allo‐SCT for MRD^low^ patients. Grouped by CD19 expression, allo‐SCT demonstrated lower CIR (*p* < 0.001) and better OS (*p* = 0.002) than CMT for CD19^−^ patients. CMT had a higher CIR (41.37% vs. 10.48%, *p* = 0.007) but comparable OS (*p* = 0.147) than allo‐SCT for CD19^+^ patients. Grouped by MRD combined with CD19, MRD^high^/CD19^+^ subsets were identified out of CD19^+^ patients benefiting from allo‐SCT with lower CIR (*p* = 0.002) and superior OS (*p* = 0.020) than CMT. CMT preserved comparable CIR (*p* = 0.939) and OS (*p* = 0.658) with allo‐SCT for MRD^low^/CD19^+^ patients. MRD^low^/CD19^−^ subsets were also identified from MRD^low^ patients requiring allo‐SCT with lower CIR (*p* < 0.001) and superior OS (*p* = 0.008) than CMT. Allo‐SCT maintained lower CIR (*p* < 0.001) and superior OS (*p* = 0.008) than CMT for MRD^high^/CD19^−^ patients.

**Conclusions:**

MRD combined with CD19 might optimize PRT choices for adult t(8;21) AML patients in CR1.

## 1 INTRODUCTION

T(8;21) acute myeloid leukemia (AML) is characterized by t(8;21)(q22;q22) chromosomal rearrangement and *RUNX1‐RUNX1T1* fusion gene, accounting for about 7% of adult AML patients. The European LeukemiaNet (ELN) Recommendations^1^ classified this AML subtype into a favorable‐risk group and recommended postremission treatment (PRT) with high‐dose cytarabine for patients in first complete remission (CR1). However, 40% of t(8;21) AML patients relapse and only approximately 60% of them could achieve long‐term survival.^2^, ^3^, ^4^, ^5^ Therefore, the PRT strategies need to be further explored for adult t(8;21) AML in CR1.

In the past, researchers have used real‐time quantitative polymerase chain reaction (RT‐qPCR) methodology to routinely monitor the *RUNX1/RUNX1T1* transcript levels as measurable residual disease (MRD). The MRD assessment in t(8;21) AML patients helps in identifying the patients with a higher relapse risk.^6^, ^7^ As a result, it was employed to guide PRT for t(8;21) AML. However, the optimal time point and the threshold levels of *RUNX1/RUNX1T1* transcripts that indicate relapse are still debatable.^9^, ^10^ Furthermore, t(8;21) AML patients with a low MRD level after chemotherapy, such as >3 log reduction of *RUNX1/RUNX1T1* transcripts in comparison to their pretreatment baseline level, exhibited a cumulative incidence of relapse (CIR) of up to 30%.^7^, ^9^ Therefore, MRD alone might not be enough for risk‐direct PRT selection. Recently, Qin et al. investigated the impact of c‐Kit mutation in combination with MRD on outcomes in t(8;21) AML.^11^ They observed that the KITD816/D820 patients with higher MRD levels after the second consolidation treatment showed the maximal 3‐year CIR. On the other hand, the patients undergoing allo‐SCT had significantly lower CIR and higher overall survival (OS) compared with those undergoing chemotherapy. They further noted that the KITD816/D820 patients with lower MRD levels, KITN822 patients, and KITexon8/WT patients with higher MRD levels displayed intermediate 3‐year CIR, while those undergoing allo‐HSCT had lower CIR but comparable OS in comparison to patients undergoing chemotherapy. It was also noted that the KITexon8/WT patients with lower MRD levels demonstrated the lowest 3‐year CIR, wherein allo‐SCT decreased both relapse and survival.^11^ Therefore, integrated evaluation of baseline prognosticator and MRD might better guide risk stratification and PRT in t(8;21) AML.

B‐cell‐specific cell‐surface CD19 antigen is frequently co‐expressed with myeloid antigens in t(8;21) AML patients.^12^, ^13^, ^14^ In the past, researchers have shown that CD19 negativity (CD19^−^) was associated with a higher relapse risk in t(8;21) AML.^15^, ^16^, ^17^ However, it is not clear whether the combination of CD19 and MRD could better guide PRT choices. In this study, we retrospectively analyzed two different postremission approaches with allo‐SCT or consolidation chemotherapy (CMT) for t(8;21) AML in CR1, to determine whether the combination of CD19 and MRD could optimize the PRT choices for t(8;21) AML patients in CR1.

## 2 METHODS

### 2.1 Patients

In this study, we screened a total of 176 recently diagnosed t(8;21) AML patients at Nanfang Hospital (Guangzhou province, China) between January 2010 and June 2021. Depending on their PRT approaches, the patients were categorized into two groups, that is, those undergoing consolidation chemotherapy (CMT) and those undergoing allo‐SCT. The CMT group included patients who had undergone at least two consolidation chemotherapy cycles and were not scheduled for upfront SCT. Furthermore, the patients in the CMT group also included the patients who had relapsed after CMT and were administered SCT subsequently. The following inclusion criteria were used for enrolling the patients: (1) aged above 14 years, (2) newly diagnosed with AML with t(8;21) and/or *RUNX1/RUNX1T1* transcripts, and (3) those who acquired CR after 1–2 induction chemotherapy cycles. On the other hand, the following exclusion criteria were implemented in this study: (1) patients who died during induction therapy, (2) those who could not achieve CR after two induction chemotherapy cycles, (3) those who received less than two consolidation cycles, and (4) the patients who did not receive any form of treatment. All the surviving patients were last followed up until December 31, 2021. The institutional review board at the Nanfang Hospital reviewed and approved the procedures implemented in this study. In accordance with the principles laid down in the Declaration of Helsinki, all the patients or their guardians were asked to provide their written informed consent before enrolling in this study.

### 2.2 Genetic and *RUNX1/* *RUNX1T1* MRD assessment

Here, we used the Giemsa and reverse banding techniques and the fluorescence in situ hybridization procedure for carrying out cytogenetic analyses. Furthermore, the molecular markers used in the study were identified using the polymerase chain reaction (PCR) and 167‐gene next‐generation sequencing (NGS) (Table S1). The bone marrow specimens were collected from the enrolled patients at different time points, that is, after induction, after each PRT cycle, every 2 months in Year 1, every 3 months in Year 2, every 4 months in Year 3, and every 6 months in Years 4 and 5.^18^ We used the real‐time quantitative reverse transcription PCR (RT‐PCR) technique for quantifying the *RUNX1/RUNX1T1* transcript levels in the samples, and the results were presented in the form of the *RUNX1/RUNX1T1/ABL* ratio.^9^ After three courses of chemotherapy, 3‐log reduction in *RUNX1/RUNX1T1* transcripts (0.1%) compared with the pretreatment baseline of 100% in our center, was used as a threshold to distinguish high MRD level (MRD^high^ ≥ 0.1%) from low MRD level (MRD^low^ < 0.1%).

### 2.3 Multiparameter flow cytometry

Immunophenotyping was performed by eight‐color flow cytometric analysis of bone marrow samples at the time of diagnosis with FACScan (Becton Dickinson, San Jose, CA., USA) after mononuclear cell enrichment by centrifugation with Ficoll‐Paque PLUS (Amersham Biosciences, Uppsala, Sweden). Dim CD45 expressors with low side scatter signals (socalled blast gate) were gated for analysis. The antibodies used included CD2, CD3, CD5, CD7, CD10, CD11b, CD11c, CD13, CD14, CD15, CD19, CD20, cytoplasmic CD22 (cCD22), CD33, CD34, CD56, CD117, HLA‐DR, MPO, nuclear terminal deoxynucleotidyl transferase (TdT), and p‐glycophorin. The data were analyzed using the CellQuest software (Becton Dickinson). CD19 was considered positive if at least 20% of blasts expressed the antigen.

### 2.4 Treatment protocols

The regimens that were used for induction therapies included the administration of either daunorubicin (60 mg/m^2^) or idarubicin (12 mg/m^2^) for three continuous days in combination with cytarabine (100 mg/m^2^) for 7 days (i.e., “3 + 7” regimen). In the case of patients who could not achieve CR after their first induction, a second induction was administered involving daunorubicin (60 mg/ m^2^) or idarubicin (10 mg/m^2^) every day, for 3 days, combined with cytarabine (2.0 g/ m^2^) every 12 h on Days 1–3 (i.e., “3 + 3” regimen), or the same regimen that was administered during the first induction cycle. After achieving the first CR, MRD^high^ patients (*RUNX1/RUNX1T1/ABL* ≥ 0.1%) after three courses of chemotherapy were recommended for allo‐HSCT, while MRD^low^ patients (*RUNX1/RUNX1T1/ABL* < 0.1%) after three courses of chemotherapy were recommended for cytarabine‐based consolidation chemotherapy. Among patients who could not comply with the treatment recommendations described above due to patient bias or donor availability, MRD^low^ patients underwent allo‐HSCT and MRD^high^ patients underwent chemotherapy as postremission treatment. The cytarabine‐based consolidation therapy included the “3 + 3” regimen and an intermediate/high‐dose of cytarabine (2–3 g/m^2^) twice each day on Days 1–3.^19^ Allo‐SCT was also advised for patients who had relapsed after CMT.^17^ The donor selection principle for allo‐SCT was based on the Chinese consensus.^20^, ^21^ All the patients received busulfan‐based myeloablative conditioning regimens.

### 2.5 Evaluation points and definition

The basic objective of this research study was to determine the 3‐year OS duration of the patients. The second endpoint included the cumulative incidence of relapse (CIR), leukemia‐free survival (LFS), nonrelapse mortality (NRM), GVHD‐free survival, and relapse‐free survival (GRFS). Complete Remission (CR) was defined as <5% blasts based on the morphologic assessment of the bone marrow (BM) samples with no clear evidence that indicates dysplasia in BM and no development of leukemia outside the hematopoietic system. Relapse was defined based on the morphologic evidence noted in the marrow, peripheral blood, or extramedullary regions.^1^ NRM was described as death without any evidence indicating the relapse of leukemia. OS was calculated from Day 1 of the therapy to death or censored at the final follow‐up. Furthermore, we determined the LFS from CR1 to relapse or death or censored at the final follow‐up. The pattern and severity of organ involvement were used to define and grade acute GVHD, while the NIH criteria were used for defining and grading chronic GVHD.^22^ Graft‐vs‐host‐disease‐free, relapse‐free survival (GRFS) events were defined as grade III or IV acute GVHD, chronic GVHD requiring systemic immunosuppressive treatment, leukemia relapse, or death from any cause from CR1 to last follow‐up. The GRFS condition presented by the patients in the chemotherapy group was regarded as equal to LFS as they did not show any incidence of GVHD and hence was compared to that exhibited by the patients in the allo‐SCT group.

### 2.6 Statistical analysis

The differences in the data among different groups were compared using the χ^2^ test or Fisher's exact test for categorical variables, whereas the Mann–Whitney U test was employed for comparing the continuous variables. The Kaplan–Meier technique was utilized for estimating the OS and LFS and compared with the log‐rank test. Cumulative incidence curves were used in a competing risk setting with relapse treated as a competing event to calculate NRM and with NRM treated as a competing risk to calculate relapse. The Cox regression model was implemented for conducting the univariate and multivariate analysis. The multivariate analysis included the variables showing *p* < 0.10 in univariate analysis or any variable affecting the outcome. A two‐sided *p*‐value of <0.05 was considered statistically significant. The data in the study were statistically analyzed using SPSS (ver. 26.0) and R (4.1.1) software.

## 3 RESULTS

### 3.1 Patient characteristics

**FIGURE 1:**
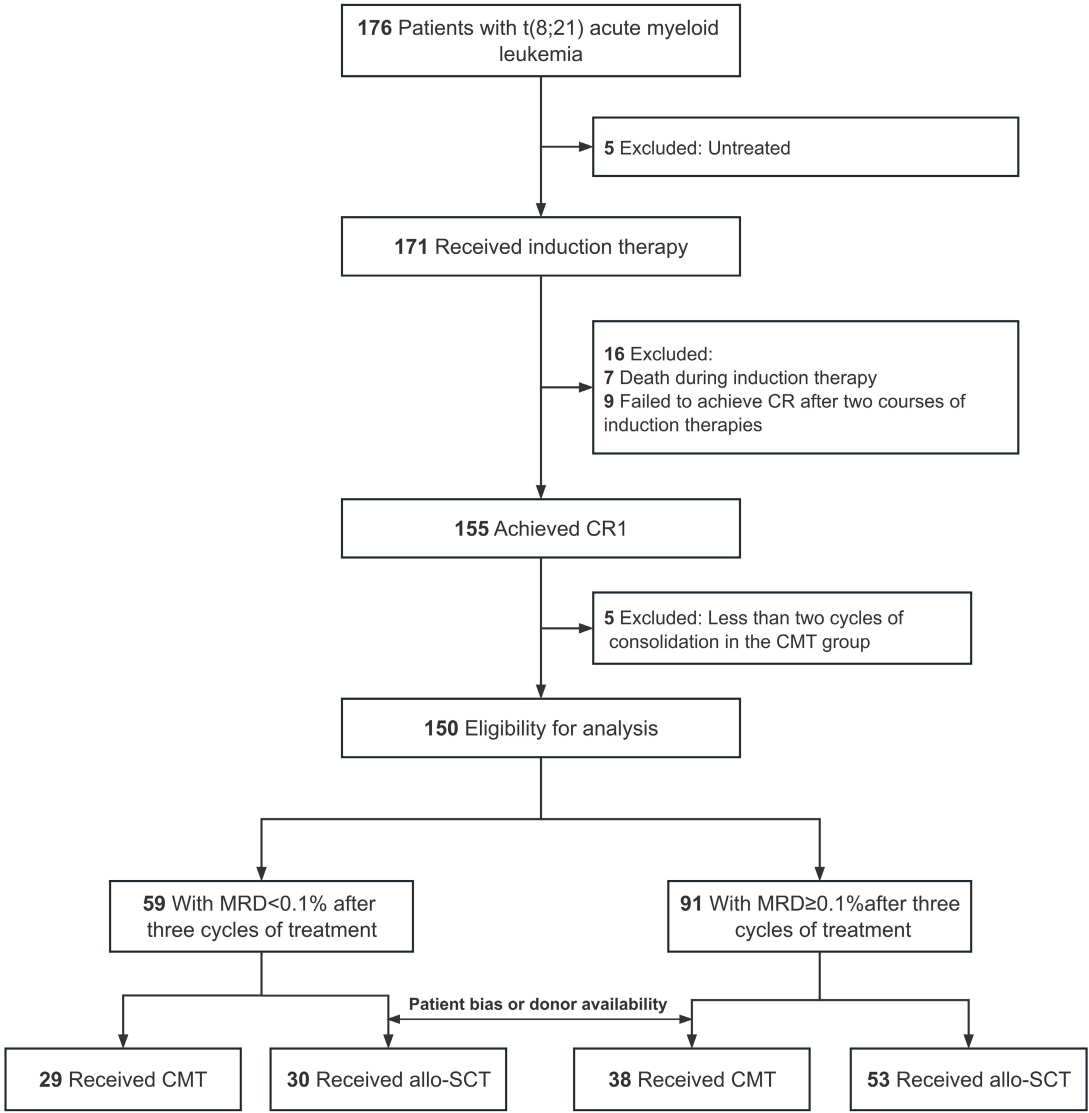
The flow diagram of t(8;21) AML patients. CR1, first complete remission; CMT, chemotherapy; Allo‐SCT, allogeneic hematopoietic stem cell transplantation.

A total of 150 patients were enrolled in this study, including 67 in the CMT and 83 in allo‐SCT groups (Figure 1 Flow diagram). In the allo‐SCT group, 37 patients were transplanted with the matched sibling donors (MSD) and 46 alternative donors (which comprised 40 haploidentical donors [HID] and 6 matched unrelated donors [MUD]). The median age was 33 (range:14–66) years, with 34 (range: 14–66) years in CMT and 32 (range: 14–61) years in allo‐SCT. Table 1 presents the characteristics of all the patients included in this study. The median percentage of blasts expressing CD19 was 36.53% (range: 20.14%–79.45%) for CD19^+^ group and 9.26% (range: 0.31%–17.18%) for CD19^−^ group (Figure S1).

**TABLE 1:** Characteristics of t(8;21)AML patients.

	Total (*n* = 150)	CMT (*n* = 67)	Allo‐SCT (*n* = 83)	*p*
Median age, y (range)	33 (14–66)	34 (14–66)	32 (14–61)	0.368
Gender (male/female), *n*	88/62	41/26	47/36	0.619
Median WBC, 10^9^/L (range)	10.80 (0.67–59.26)	10.03 (1.44–587)	12.40 (0.67–59.26)	0.451
Median BM blasts, % (range)	43.0 (5.0–94.0)	43.66 (5.0–94.0)	43.0 (7.5–94)	0.274
AML type, *n* (%)				
De novo	145 (96.7)	64 (95.5)	81 (97.6)	0.484
Secondary	5 (3.3)	3 (4.5)	2 (2.4)	
Extramedullary involvement, *n* (%)	14 (9.3)	8 (11.9)	6 (7.2)	0.324
CD19 expression, *n* (%)	75 (56.4)	24 (42.1)	51 (67.1)	0.004
CD33 expression, *n* (%)	108 (81.8)	44 (78.6)	64 (84.2)	0.406
CD56 expression, *n* (%)	84 (63.6)	32 (57.1)	52 (68.4)	0.183
Additional cytogenetic abnormalities, *n* (%)	53 (49.1)	21 (50.0)	32 (48.5)	0.878
Loss of X/Y, *n* (%)	39 (36.1)	17 (40.5)	22 (33.3)	0.451
≥3 additional cytogenetic abnormalities, *n* (%)	5 (6.0)	2 (4.8)	3 (7.1)	0.644
*KIT*, *n* (%)	54 (45.0)	19 (40.4)	35 (47.9)	0.419
*KIT* exon 8, *n* (%)	5 (4.2)	2 (4.3)	3 (4.1)	0.969
*KIT* exon 17, *n* (%)	48 (40.0)	19 (40.4)	29 (39.7)	0.939
*KIT D816*, *n* (%)	34 (28.3)	14 (29.8)	20 (27.4)	0.777
*KIT N822*, *n* (%)	12 (10.0)	4 (8.5)	8 (11.0)	0.660
*FLT3*, *n* (%)	24 (20.0)	10 (21.3)	14 (19.2)	0.779
*FLT3‐ITD, n* (%)	14 (11.7)	5 (10.6)	9 (12.3)	0.778
*FLT3‐TKD*, *n* (%)	11 (9.2)	4 (8.5)	7 (9.5)	0.842
*TET2, n* (%)	50 (41.7)	21 (44.7)	29 (39.7)	0.591
MRD^low^	59 (39.3)	29 (43.3)	30 (36.1)	0.374
No. of cycles to CR				
One	114 (76.0)	47 (70.1)	67 (80.7)	0.132
Two	36 (24.0)	20 (29.9)	16 (19.3)	
Outcome				
Relapse, *n* (%)	44 (29.3)	35 (52.2)	9 (10.8)	<0.001
Death, *n* (%)	31 (20.7)	19 (28.4)	12 (14.5)	0.037
NRM, *n* (%)	10 (6.7)	2 (3.0)	8 (9.6)	0.091
3–4 grade GVHD, *n* (%)	–	–	17 (20.5)	–
CNSL, *n* (%)	8 (5.3)	3 (4.5)	5 (6.0)	0.673

Abbreviations: AML, acute myeloid leukemia; Allo‐SCT, allogeneic hematopoietic stem cell transplantation; BM, bone marrow; CR, complete remission; CMT, chemotherapy; CNSL, central nervous system leukemia; GVHD, graft versus‐host disease; MRD^low^, a > 3‐log reduction in RUNX1/RUNX1T1 transcripts after three courses of chemotherapy; NRM, nonrelapse mortality; WBC, white blood cells.

### 3.2 Relapse and survival

At the last follow‐up, 44 patients relapsed. Of the 35 patients who relapsed in the CMT group, 12 received allo‐SCT and 19 received salvage chemotherapy. However, 14 survived and 17 died at the last follow‐up. Of the 9 patients who relapsed in allo‐SCT group, 8 received salvage treatment, 3 were still alive and 5 were died at the last follow‐up. For the entire study population, the 3‐year CIR was 32.91%.Patients undergoing allo‐SCT had a significantly lower CIR compared with those in the CMT group (12.08% vs. 59.81%, *p* < 0.001). Even the results of the multivariate analysis indicated that the allo‐SCT patients exhibited significantly lower CIR compared to CMT patients (HR, 0.34 [95% CI, 0.15–0.75]; *p* = 0.008). MRD^high^ after three courses of treatment (HR, 0.14 [95% CI, 0.03–0.66]; *p* = 0.013), KIT 816 mutation (HR, 6.73 [95% CI, 4.47–8.78]; *p* = 0.012) and CD19 negativity (HR, 0.14 [95% CI, 0.02–0.96]; *p* = 0.045) were identified as the independent risk factor for relapse (Table 2). Therefore, postremission treatment selection was investigated based on MRD level after three course of treatment as well as in combination with CD19 expression at diagnosis across the entire cohort. The CMT group and allo‐SCT groups showed 3‐year NRM values of 3.09% and 9.36%, respectively (*p* = 0.118). With a median follow‐up of 36 months (range: 3–142 months), 101 patients were still alive, while 31 patients died. The complete study population showed the 3‐year OS and 3‐year LFS of 75.79% and 61.66%, respectively. The patients in the CMT and allo‐SCT groups showed a 3‐year OS of 64.73% and 83.36%, respectively (*p* = 0.008). Additionally, the patients in CMT and allo‐SCT groups showed 3‐year LFS of 38.78% and 79.03%, respectively (*p* < 0.001). Multivariate analysis indicated that allo‐SCT exhibited a better LFS (HR, 0.30 [95% CI, 0.14–0.66]; *p* = 0.003) and OS (HR, 0.30 [95% CI, 0.11–0.86]; *p* = 0.025) than CMT. Furthermore, KIT 816 mutation was identified as the risk factor for LFS (HR, 8.65 [95% CI, 5.54–11.34]; *p* = 0.013) and OS (HR, 3.42 [95% CI, 1.20–9.76]; *p* = 0.022) in multivariate analysis (Table 2).

**TABLE 2:** Univariate and multivariate analysis for OS, CIR, and LFS.

	OS		CIR		LFS	
	Univariate HR (95% CI); *p*	Multivariate HR (95% CI); *p*	Univariate HR (95% CI); *p*	Multivariate HR (95% CI); *p*	Univariate HR (95% CI); *p*	Multivariate HR (95% CI); *p*
Age (≥33 vs. <33 years)	0.92 (0.47–1.78); 0.793	–	0.52 (0.27–1.02); 0.060	2.15 (0.38–12.13); 0.385	0.89 (0.53–1.50); 0.657	–
Gender (male vs. female)	1.53 (0.78–2.99); 0.237	–	1.48 (0.76–2.88); 0.263	–	1.52 (0.90–2.57); 0.126	–
WBC count (≥10.8 vs. <10.8 × 10^9/L)	1.61 (0.83–3.13); 0.164	–	1.40 (0.72–2.75); 0.322	–	1.53 (0.88–2.60); 0.117	–
BM blasts (≥43% vs. <43%)	1.25 (0.64–2.47); 0.505	–	1.09 (0.56–2.12); 0.789	–	1.18 (0.70–2.01); 0.526	–
Extramedullary involve (yes vs. no)	2.48 (0.79–7.84); 0.024	1.39 (0.30–6.48); 0.71	1.68 (0.58–4.87); 0.233	–	1.46 (0.62–3.45); 0.314	–
Mutated KIT 816 (positive vs. negative)	2.78 (0.92–8.44); 0.021	3.42 (1.20–9.76); 0.022	4.01 (1.35–11.95); <0.001	6.73 (4.47–8.78); 0.012	2.06 (1.01–4.72); 0.039	8.65 (5.54–11.34); 0.013
Cycles to achieve CR (two vs. one)	1.75 (0.72–4.30); 0.142	–	2.71 (1.06–6.96); 0.004	1.27 (0.19–8.44); 0.807	1.90 (1.04–4.20); 0.047	0.51 (0.04–6.78); 0.612
Allo‐SCT versus CMT	0.34 (0.16–0.71); <0.001	0.30 (0.11–0.86); 0.025	0.32 (0.15–0.67); <0.001	0.34 (0.15–0.75); 0.008	0.37 (0.21–0.66); <0.001	0.30 (0.14–0.66); 0.003
CD19 (positive vs. negative)	0.52 (0.25–1.11); 0.077	0.60 (0.22–1.64); 0.323	0.37 (0.19–0.74); 0.004	0.14 (0.02–0.96); 0.045	0.46 (0.25–0.84); 0.011	1.15 (0.20–6.75); 0.876
MRD1 (<0.1% vs. ≥0.1%)	1.16 (0.21–6.32); 0.859	–	0.80 (0.18–3.53); 0.779	–	1.25 (0.36–4.28); 0.713	–
MRD2 (<0.1% vs. ≥0.1%)	0.80 (0.17–3.71); 0.774	–	1.97 (0.51–7.70); 0.385	–	1.31 (0.43–4.06); 0.646	–
MRD3 (<0.1% vs. ≥0.1%)	0.35 (0.11–1.16); 0.107	–	0.37 (0.12–1.10); 0.082	0.14 (0.03–0.66); 0.013	0.37 (0.17–0.82); 0.019	0.22 (0.03–1.50); 0.122
MRD4 (<0.1% vs. ≥0.1%)	0.82 (0.22–3.01); 0.760	–	0.56 (0.14–2.24); 0.419	–	0.62 (0.24–1.55); 0.306	–

Abbreviations: Allo‐SCT, allogeneic hematopoietic stem cell transplantation; BM, bone marrow; CMT, chemotherapy; CR, complete remission; CIR, cumulative incidence of relapse; LFS, leukemia‐free survival; OS, overall survival; WBC, white blood cells.

### 3.3 PRT selection and outcomes based on MRD

**FIGURE 2:**
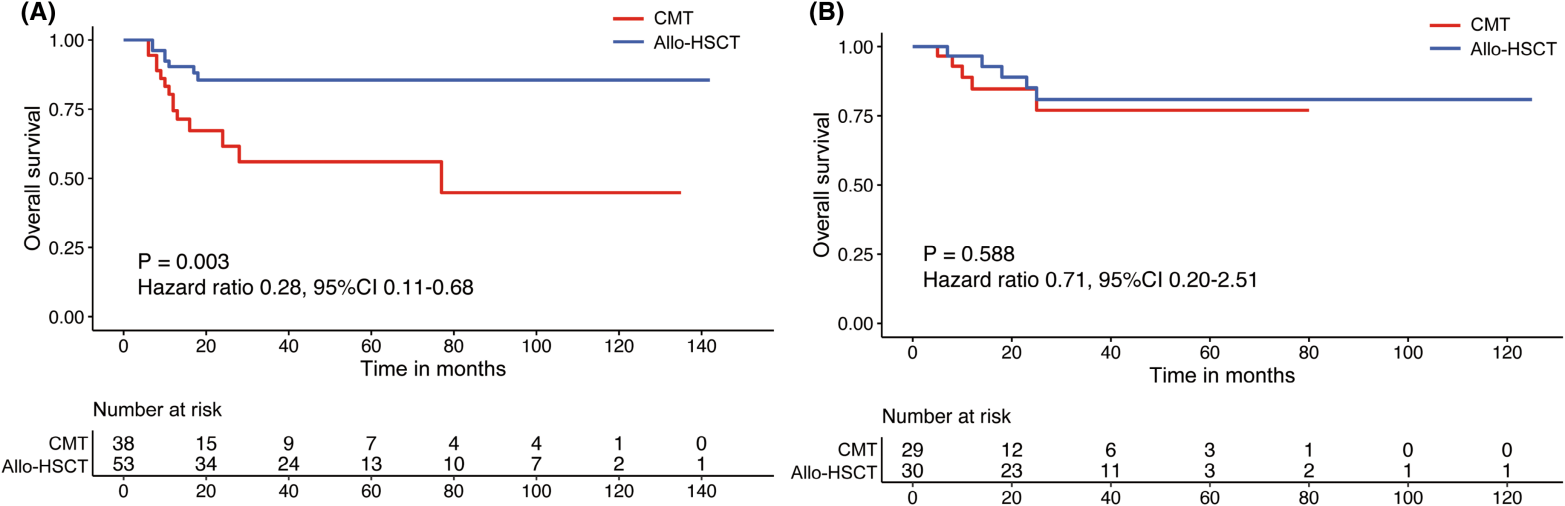
Overall survival (OS) according to MRD after three courses of chemotherapy. (A) MRD^high^ group; (B) MRD^low^ group. CMT, chemotherapy; Allo‐SCT, allogeneic hematopoietic stem cell transplantation; MRD^high^, *RUNX1/RUNX1T1* transcripts >0.1% after three courses of chemotherapy; MRD^low^, *RUNX1/RUNX1T1* transcripts <0.1% after three courses of chemotherapy.

In the case of t(8;21) AML patients with MRD^high^ after three cycles of treatment, it was noted that the patients in the allo‐SCT group displayed a significantly lower 3‐year CIR compared to the patients in CMT (10.03% vs. 74.31%, *p* < 0.001). They further exhibited better LFS in comparison to the CMT group (82.11% vs. 24.90%, *p* < 0.001). Furthermore, the Allo‐SCT group also showed better 3‐year GRFS (52.56% vs. 24.90%, *p* = 0.009) and 3‐year OS (85.58% vs. 56.03%, *p* = 0.003) than the CMT patients (Figure 2A). On the contrary, in the case of MRD^low^ patients after three cycles of treatment, those in the allo‐SCT preserved a trend toward lower 3‐year CIR (15.34% vs. 35.99%, *p* = 0.100) compared to the CMT group. However, patients in allo‐SCT and CMT groups exhibited similar 3‐year LFS (74.35% vs. 61.55%, *p* = 0.274), 3‐year GRFS (52.87% vs. 61.55%, *p* = 0.576), and 3‐year OS (80.85% vs. 77%, *p* = 0.588) values (Figure 2B). This study also indicated allo‐SCT could reduce the relapse rate and improve the survival duration of the MRD^high^ patients compared to those receiving CMT treatment. However, the 3‐year CIR remained at 35.99% in MRD^low^ patients undergoing CMT.

### 3.4 PRT selection and outcomes based on CD19 expression

**FIGURE 3:**
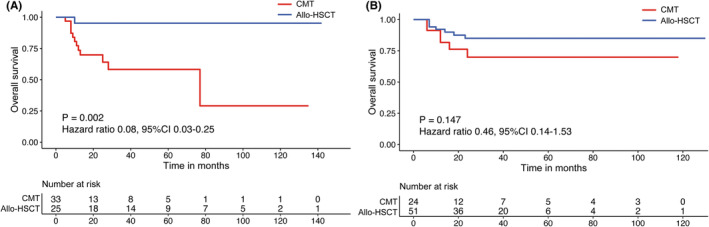
Overall survival (OS) according to CD19 expression. (A) CD19^−^ group (B) CD19^+^ group. CMT, chemotherapy; Allo‐SCT, allogeneic hematopoietic stem cell transplantation; CD19^−^, CD19 negativity; CD19^+^, CD19 positivity.

We further investigated the association of CD19 expression with PRT choice and outcomes. It was observed that in the case of CD19^−^ patients, allo‐SCT significantly decreased 3‐year CIR compared to CMT (9.32% vs. 66.25%, *p* < 0.001), thereby exhibiting better LFS than CMT (87.05% vs. 32.50%, *p* < 0.001). Allo‐SCT also showed better 3‐year GRFS (54.40% vs. 32.50%, *p* = 0.059) and 3‐year OS (95.24% vs. 58.24%, *p* = 0.002) than those shown by CMT (Figure 3A). On the contrary, in the case of CD19^+^ patients, allo‐SCT yielded a lower 3‐year CIR (10.48% vs. 41.37%, *p* = 0.007) and better 3‐year LFS (81.26% vs. 55.84%, *p* = 0.035) compared to those undergoing CMT. However, this did not translate into a better OS. The allo‐SCT and CMT groups showed similar 3‐year GRFS (57.82% vs. 55.84%, *p* = 0.918) and 3‐year OS (85.03% vs. 69.89%, *p* = 0.147) (Figure 3B). Thus, the findings in this study indicated that allo‐SCT could decrease relapse rate and improve the survival duration of CD19^−^ patients in comparison to CMT. However, the 3‐year CIR remained at 41.37% in CD19^+^ patients undergoing CMT.

### 3.5 PRT selection and outcomes based on MRD in combination with CD19

**FIGURE 4:**
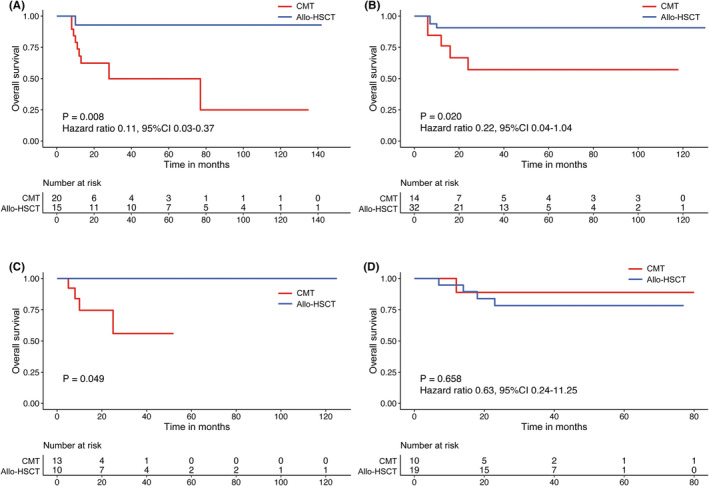
Overall survival (OS) according to MRD combined with CD19 expression. (A) MRD^high^/CD19^−^ group, (B) MRD^high^/CD19^+^ group, (C) MRD^low^/CD19^−^ group, and **(D)** MRD^low^/CD19^−^ group. CMT, chemotherapy; Allo‐SCT, allogeneic hematopoietic stem cell transplantation; MRD^high^, *RUNX1/RUNX1T1* transcripts >0.1% after three courses of chemotherapy; MRD^low^, *RUNX1/RUNX1T1* transcripts <0.1% after three courses of chemotherapy; CD19^−^, CD19 negativity; CD19^+^, CD19 positivity.

Finally, we investigate the association of integrated results of CD19 and MRD after three cycles of treatment, PRT selection, and outcomes. In this study, 133 patients were further categorized into four subgroups: (I) subgroup A, MRDhigh/CD19‐; (II) subgroup B, MRDhigh/CD19+; (III) subgroup C, MRDlow/CD19‐; and (IV) subgroup D, MRDlow/CD19+. The patient's characteristics among the four groups are shown in Table 3. The patients in subgroup A undergoing allo‐SCT showed a lower 3‐year CIR than those undergoing CMT (7.69% vs. 78.75%, *p* < 0.001). They also showed better 3‐year LFS than those undergoing CMT (86.15% vs. 21.25%, *p* < 0.001), better 3‐year GRFS (50% vs. 21.25%, *p* = 0.060), and 3‐year OS (92.86% vs. 49.88%, *p* = 0.008) than CMT (Figure 4A). Furthermore, the patients in subgroup B undergoing allo‐SCT also demonstrated a lower 3‐year CIR compared with those undergoing CMT (9.48% vs. 57.44%, *p* = 0.002) as well as better 3‐year LFS (87.50% vs. 38.69%, *p* = 0.001), 3‐year GRFS (62.20% vs. 38.69%, *p* = 0.210), and 3‐year OS (90.63% vs. 57.12%, *p* = 0.020) (Figure 4B). The patients in subgroup C, who received allo‐SCT showed a lower 3‐year CIR (12.50% vs. 70.09%, *p* = 0.028), higher 3‐year LFS (87.50% vs. 26.92%, *p* = 0.017), and improved 3‐year GRFS (62.50% vs. 26.92%, *p* = 0.106) in comparison to CMT, leading to a superior OS (100% vs. 55.94%, *p* = 0.049) than CMT (Figure 4C). In subgroup D, the CMT and allo‐SCT groups showed comparable 3‐year CIR (10% vs. 11.85%, *p* = 0.939), 3‐year LFS (88.89% vs. 72.55%, *p* = 0.476), and 3‐year OS (90% vs. 78.92%, *p* = 0.658) (Figure 4D). Also, it was seen that CMT improved their 3‐year GRFS (88.89% vs. 52.11%, *p* = 0.098) than allo‐SCT. Thus, the findings presented in this study implied that allo‐SCT could be recommended as a treatment strategy for patients in subgroups A, B, and C as they showed a better OS than CMT, whereas CMT could be recommended for subgroup D patients as they showed a comparable OS with allo‐SCT.

**TABLE 3:** Characteristics patients based on MRD and CD19 status.

	MRD^high^CD19^−^ (*n* = 35)	MRD^high^CD19^+^ (*n* = 46)	MRD^low^CD19^−^ (*n* = 23)	MRD^low^CD19^+^ (*n* = 29)
Median age, years (range)	32 (16–50)	35 (15–58)	42 (14–59)	35 (14–66)
Gender (male/female), *n*	25/10	29/17	16/7	16/13
Median WBC, 10^9^/L (range)	11.4 (4.53–56.04)	10.07 (2.10–59.26)	9.06 (2.70–16.39)	7.34 (0.67–51.74)
Median BM blasts, % (range)	38.5 (20.5–94.0)	30.0 (5.0–90.5)	43.7 (25.0–78.5)	39.5 (15.5–82.5)
AML type, *n* (%)				
De novo	35 (100)	46 (100)	21 (91.3)	28 (96.6)
Secondary	0	0	2 (8.7)	1 (3.4)
Extramedullary involvement, *n* (%)	2 (5.7)	3 (6.5)	4 (17.4)	3 (10.3)
Additional cytogenetic abnormalities, *n* (%)	15 (42.9)	18 (39.1)	10 (43.5)	9 (31.0)
Loss of X/Y, *n* (%)	10 (28.6)	14 (30.4)	6 (26.1)	7 (24.1)
≥3 additional cytogenetic abnormalities, *n* (%)	0	1 (2.2)	1 (4.3)	0
*KIT*, *n* (%)	15 (42.9)	19 (41.3)	9 (39.1)	10 (34.5)
*KIT* exon 8, *n* (%)	1 (2.8)	1 (2.2)	0	1 (3.4)
*KIT* exon 17, *n* (%)	13 (37.1)	16 (34.8)	7 (30.4)	9 (31.0)
No. of cycles to CR				
One	25 (71.4)	36 (78.3)	19 (82.7)	23 (79.3)
Two	10 (28.6)	10 (21.7)	4 (17.4)	6 (20.7)

*Note*: *, **p values <0.05 between each pair indicated in rows.

Abbreviations: BM, bone marrow; CR, complete remission; CD19^+^, CD19 positivity; CD19^−^, CD19 negativity; MRD^high^, RUNX1/RUNX1T1 transcripts >0.1% after three courses of chemotherapy; MRD^low^, RUNX1/RUNX1T1 transcripts <0.1% after three courses of chemotherapy; WBC, white blood cells.

To further figure out whether the predicted power of MRD combined with CD19 was superior to MRD alone for outcomes in t(8;21) AML patients, we utilized time‐dependent ROC and Harrell's Harmony Index (C‐index). Based on the prognosis‐related independent factors screened by the multivariate Cox regression, we constructed an OS‐related model that included MRD alone or MRD combined with CD19, namely MRD model and MRD/CD19 model. Compared with the MRD model, the MRD/CD19 model had a higher C‐index of predicting overall survival (OS) (0.756 vs. 0.671, *p* = 0.010). For MRD and MRD/CD19 models, the AUC values of 3‐year OS were 0.723 and 0.804, 5‐year OS were 0.640 and 0.840, respectively (Supplemental figure2). These results suggest that MRD combined with CD19 was more effective than CD19 alone predicting survival.

## 4 DISCUSSION

To our knowledge, this study first demonstrated MRD in combination with CD19 might better guide PRT choices compared with MRD or CD19 alone for t(8;21)AML in CR1. The results showed that for MRD^high^/CD19^−^, MRD^high^/CD19^+^, and MRD^low^/CD19^−^ patients, allo‐SCT might be recommended in CR1; for MRD^low^/CD19^+^ patients, CMT might be recommended.

Optimizing postremission therapy for t(8;21) AML patients remains a major clinical challenge. Several studies have concluded that MRD persistence was related to an increasing relapse risk, resulting in poor outcomes in t(8;21) AML patients.^9^, ^10^, ^23^ This provides a strong rationale for employing MRD to direct PRT choices.^24^, ^25^, ^26^ In their studies, Yin et al. and Rücker et al. reported that <3 log reduction of *RUNX1/RUNX1T1* transcripts in comparison to the pretreatment baseline values after three courses of chemotherapy indicated a higher relapse risk.^6^, ^9^ We recently reported that the favorable‐risk MRD‐positive patients after three chemotherapy cycles or recurrent MRD showed higher CIR and benefited from allo‐SCT.^19^ In this study, MRD^high^ after three courses of treatment was identified as an independent risk factor for relapse. Hence, MRD level after three cycles of treatment was employed to assess PRT choice. Similar to the results reported earlier,^8^ we also noted that the t(8;21) patients with MRD^high^ after three courses of treatment benefited significantly from allo‐SCT and showed lower CIR and better OS values compared to those undergoing CMT. However, in the case of MRD^low^ patients who were administered three courses of treatment, chemotherapy showed a higher CIR of 35.99%. This finding was similar to the value reported earlier, that is, 30%.^7^, ^9^ In addition to MRD itself, unfavorable molecular prognostic factors may also affect the rate of relapse in this population. Although allo‐SCT tends to decrease relapse when compared to consolidation chemotherapy for MRD^low^ patients after three courses of treatment, the OS and GRFS values were similar in both groups. This could be attributed to the fact that allo‐SCT could be used as a salvage treatment strategy after relapse, which was consistent with the findings published in the past.^27^, ^28^ However, some reports showed that the patients who were administered allo‐SCT in CR2 did not show better OS and LFS compared to those transplanted in CR1.^29^, ^30^, ^31^ This gives rise to the question: whether it is possible to identify the subsets in t(8;21) AML patients with low MRD levels that could benefit from allo‐SCT in CR1 by integrating other prognostic factors with MRD.

Numerous factors exert an impact on clinical outcomes of t(8;21) AML. These factors mainly consist of cytogenetic and molecular alterations, such as co‐occurring gene mutations of tyrosine kinase pathway (such as KIT, N/KRAS, and FLT3), epigenetic regulators and cohesin complex, and additional chromosomal abnormalities.^32^, ^33^ However, their prognostic significance remained debatable. For example, loss of a sex chromosome,^34^, ^35^ additional three or more chromosomal abnormalities,^2^, ^36^ and KIT mutations, have been differently reported.^37^, ^38^ It has been noted that the t(8;21) AML patients frequently expressed the B‐cell marker CD19 (ranging from 36% to 75%) with myeloid antigens in their myeloblasts.^12^, ^13^, ^14^ Negative CD19 expression is associated with KIT‐activating mutations in t(8;21) AML,^39^ and was related to a higher relapse risk in t(8;21) AML patients.^15^, ^16^, ^17^ Similarly, our study also revealed that the CD19^−^ patients showed a higher CIR than CD19^+^ patients. To date, there have been no reports investigating the impact of CD19 on PRT choices for t(8;21) AML patients. In our study, we observed that allo‐SCT significantly decreased the relapse rate and improved the survival duration for CD19^−^ patients compared with consolidation chemotherapy. For CD19+ patients, allo‐SCT had higher LFS than CMT owing to significantly lower CIR in the former, indicating that allo‐SCT exerts a stronger anti‐leukemia effect than CMT for this subset. However, OS and GRFS were comparable between the two groups. Notably, the 3‐year CIR remained 41.37% in CD19^+^ patients undergoing CMT as PRT, indicating CD19 alone might be insufficient to guide PRT selection.

Several studies have demonstrated that the combination of cytogenetic classification and MRD status could help AML patients in CR1 make better PRT choices. Favorable‐risk and intermediate‐risk patients with high MRD levels benefited from more intensified treatment including allo‐SCT.^40^, ^41^ Qin et al. observed that the integrated evaluation of C‐Kit and MRD could better guide risk stratification and PRT in t(8;21) AML patients.^11^ This study attempted to explore whether the combination of MRD and CD19 was superior to MRD or CD19 alone in guiding PRT. In comparison to CD19 alone, CD19 combined with MRD identified MRD^high^/CD19^+^ subsets out of CD19^+^ patients that benefited from allo‐SCT. CMT preserved its advantage over allo‐SCT for MRD^low^/CD19^+^ patients. Furthermore, in comparison to MRD alone, CD19 combined with MRD also identified MRD^low^/CD19^−^ subsets out of MRD^low^ patients that required allo‐SCT treatment. Allo‐SCT maintained its superiority over CMT for MRD^high^/CD19^−^ patients. Therefore, we concluded that CD19 combined with MRD helps optimize PRT choices for t(8;21) AML in CR1.

The lower NRM (9.6%) of allo‐SCT group in this study was comparable with our former studies (10.4% and 11.6%, respectively)^19^, ^42^ and others' reports (9%).^43^ This improvement likely results from the accumulation of many individually incremental advances in conditioning therapy, GVHD prophylaxis, modern antiviral and antifungal prophylaxis, infection control, and supportive care.^44^ The lower median age of 32 years and favorable‐risk patient selection in this study might also contribute to the low rate of nonrelapse mortality.

To conclude, we noted that MRD combined with CD19 might better guide PRT choices than MRD or CD19 alone for t(8;21) AML patients in CR1. As a limited number of patients were included in the different subgroups, the results have to be explained cautiously. Additionally, the retrospective bias was inevitable. In the future, we will conduct a multicenter, prospective clinical trial to address the above issues and validate the findings noted above.

## AUTHOR CONTRIBUTIONS

**Xi Jia:** Formal analysis (equal); writing – original draft (equal). **Naying Liao:** Formal analysis (equal); writing – original draft (equal); writing – review and editing (equal). **Sijian Yu:** Methodology (equal). **Huan Li:** Resources (equal). **Hui Liu:** Resources (equal). **Haiyan Zhang:** Resources (equal). **Jun Xu:** Resources (equal). **Yunqian Yao:** Resources (equal). **Han He:** Resources (equal). **Guopan Yu:** Resources (equal). **Qifa Liu:** Conceptualization (equal); data curation (equal); supervision (equal). **Yu Zhang:** Conceptualization (equal); supervision (equal); writing – review and editing (equal). **Pengcheng Shi:** Conceptualization (equal); funding acquisition (equal); supervision (equal); writing – review and editing (equal).

## FUNDING INFORMATION

This work was supported by the Natural Science Foundation of Guangdong Province (grant no. 2020A1515010406) and the National Natural Science Foundation of China (grant no. 82370152).

## CONFLICT OF INTEREST STATEMENT

The authors declare that they have no competing interests.

## ETHICS STATEMENT

This study was approved by the institutional review boards at Nanfang Hospital. Written informed consent was obtained from all patients or guardians before entry into the study in accordance with the Declaration of Helsinki.

## CONSENT FOR PUBLICATION

Not applicable.

## Supporting information


**Figure S1.** The percentage of CD19 expression in patients grouped by CD19^−^ or CD19^+^. CD19^−^, CD19 negativity; CD19^+^, CD19 positivity.

**Figure S2.** The ROC curves to predict 3‐year and 5‐year overall survival rates in the whole t(8;21) AML patient cohort based on MRD or MRD combined with CD19. (a) MRD model, (**b**) MRD/CD19 model. MRD, measurable residual disease; ROC, receiver operating characteristic.

**Table S1.** The 167‐gene next‐generation sequencing.

## Data Availability

The data that support the findings of this study are available on request from the corresponding author. The data are not publicly available due to privacy or ethical restrictions.
