# Supplementary figures and images for: Impact of measurable residual disease in combination with CD19 on postremission therapy choices for adult t(8;21) acute myeloid leukemia in first complete remission
Source: Cancer Med. 2024 Mar 8;13(4):e7074. doi: 10.1002/cam4.7074 (PMC10922018; doi:10.1002/cam4.7074)

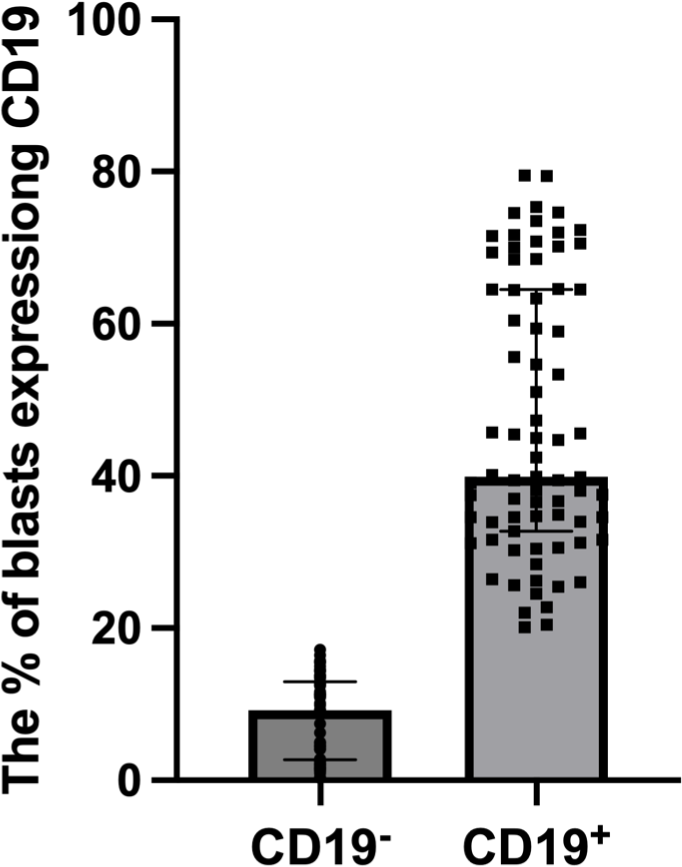

Supplement: Supplementary file 1 — Figure S1. The percentage of CD19 expression in patients grouped by CD19− or CD19+. CD19−, CD19 negativity; CD19+, CD19 positivity. [file CAM4-13-e7074-s001.tif]

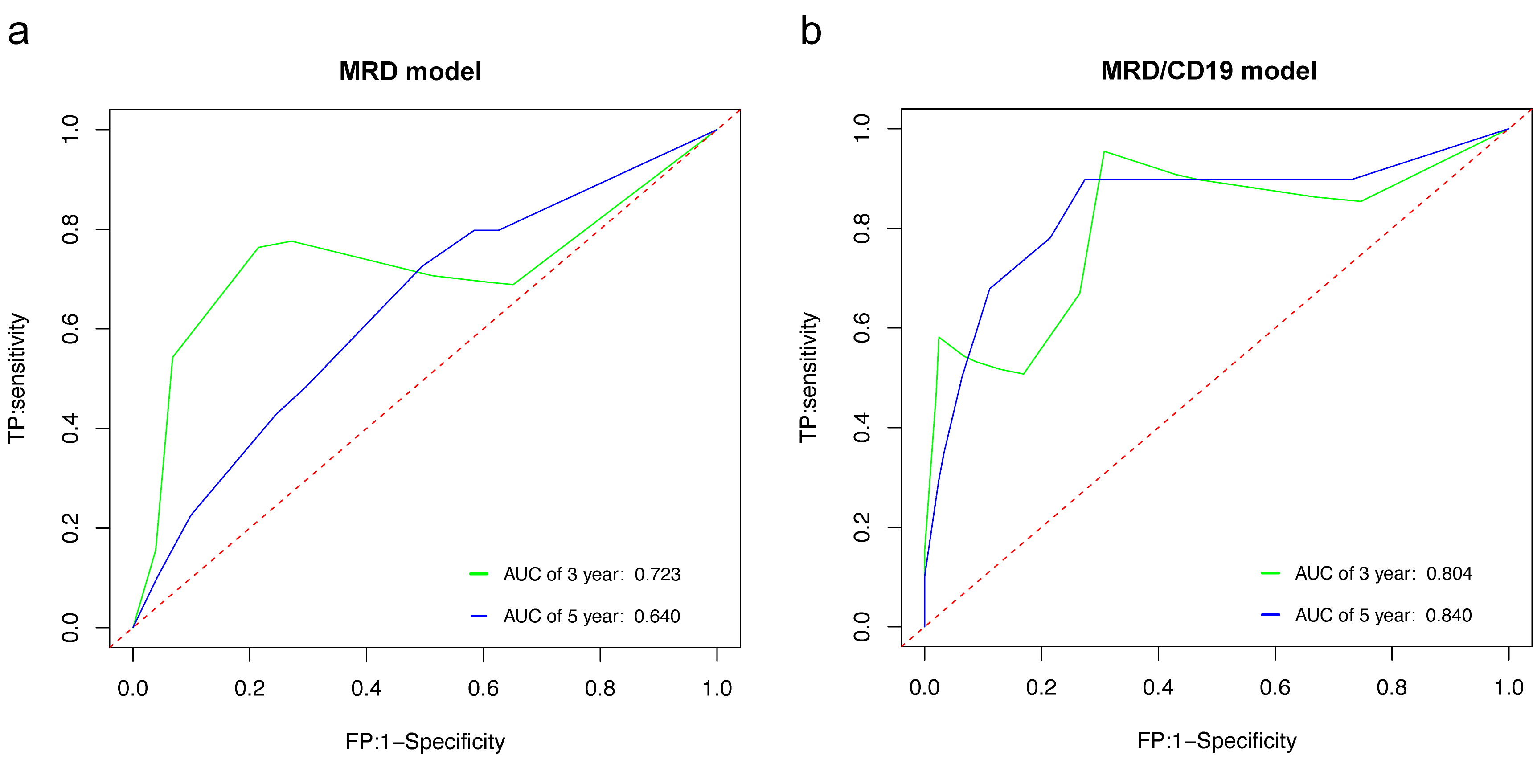

Supplement: Supplementary file 2 — Figure S2. The ROC curves to predict 3‐year and 5‐year overall survival rates in the whole t(8;21) AML patient cohort based on MRD or MRD combined with CD19. (a) MRD model, (b) MRD/CD19 model. MRD, measurable residual disease; ROC, receiver operating characteristic. [file CAM4-13-e7074-s002.tif]
