# Supplementary material for: Impact of measurable residual disease in combination with CD19 on postremission therapy choices for adult t(8;21) acute myeloid leukemia in first complete remission
Source: Cancer Med. 2024 Mar 8;13(4):e7074. doi: 10.1002/cam4.7074 (PMC10922018; doi:10.1002/cam4.7074)
Supplement: Supplementary file 3 — Table S1. The 167‐gene next‐generation sequencing. [file CAM4-13-e7074-s003.docx]

Table S1. **The 167-gene next generation sequencing.**

NGS was performed using amplicon sequencing. Primers were designed and synthesized by ThermoFisher. The Ion AmpliSeqTM Library Kit was used for library construction, and the initial template size of PCR was 12-16 ng. The Ion library TaqMan quantification kit was used for real-time quantitative PCR detection of the library. The Ion 318TM OT2 kit was used for emulsion PCR and ISP enrichment and then the Ion 318 Chip kit was applied for paired-end sequencing. The Torrent Suite software was used for primary data analysis and generating of the BAM and VCF files for each sample, and also data including the loading density, percentage of mapped reads, total amount of data and mean sequence read length of each chip, and the on-target rate, average depth and uniformity of each sample. Finally, the VCF file of each sample was annotated and visualized using the IGV software to rule out false positives (on-target rate, 97%-99%; average depth, 1000x; uniformity, 94%-97%). The Human Genome Assembly GRCh37 was used as the reference sequence. As for sensitivity, genetic variations with a VAF ≥ 5% could be detected with high reliability. After ruling out false positives using the IGV software, the rest of the genetic variations were considered positive. The 167 gene are as follows.

| ABCA12 | CBL | CYLD | FAT4 | JAK3 | MYC | PRPF40B | SRSF2 | UBA2 |
| --- | --- | --- | --- | --- | --- | --- | --- | --- |
| ABL1 | CCND1 | DDB1 | FBXW7 | KDM2B | MYD88 | PTEN | SSPO | WHSC1 |
| ABL2 | CCND3 | DDX18 | FGFR3 | KDM5A | MYH11 | PTPN11 | STAG1 | WT1 |
| ACTR5 | CD101 | DDX3X | FLT3 | KDM6A | NF1 | PU.1 | STAG2 | XBP1 |
| AKT1 | CD123 | DIS3 | GATA1 | KIT | NFKB2 | RAD21 | STAT3 | XPO1 |
| ALK | CD79b | DNM2 | GATA2 | KRAS | NOTCH1 | RB1 | STAT5a | ZEB2 |
| ANK3 | CDC27 | DNMT1 | GATA3 | LMO1 | NOTCH2 | RELN | STAT5b | ZRSR2 |
| ARID1A | CDK4 | DNMT3A | GNAS | LMO2 | NPM1 | RHOA | SUZ12 |  |
| ARID2 | CDKN2A | EBF1 | HMGA2 | MAP2K1 | NRAS | ROS1 | SYK |  |
| ASXL1 | CDKN2B | ECT2L | HMGB1 | MDM2 | NT5C2 | RUNX1 | TAL1 |  |
| ATM | CEBPA | EED | HRAS | MEF2B | NUP98 | RUNX2 | TCF3 |  |
| ATRX | CHD1 | EGFR | ID2 | MLL | PAX5 | SAMHD1 | TERC |  |
| BAFF | CRBN | EP300 | IDH1 | MLL2 | PDGFRA | SETBP1 | TET1 |  |
| BCL2 | CREBBP | EPHA7 | IDH2 | MLL3 | PDGFRB | SETD2 | TET2 |  |
| BCL6 | CRLF2 | ERG1 | IKZF | MLL5 | PHF6 | SF1 | TIM-3 |  |
| BCOR | CSF1R | ETV6 | IL7R | MMD2 | PI3KCA | SF3A1 | TLX3 |  |
| BIRC3 | CSF3R | EVI1 | IRF4 | MN1 | PICALM | SF3B1 | TNFAIP3 |  |
| BRAF | CUX1 | EZH2 | IRF6 | MPL | PKM2 | SH2B3 | TP53 |  |
| CALR | CXCL12 | FAM46C | JAK1 | MTAP | PRDM1 | SOCS1 | TRAF3 |  |
| CARD11 | CXCR4 | FAT1 | JAK2 | MUM1 | PRMT5 | SOX4 | U2AF1 |  |
